# Electrochemical Characteristics and Transport Properties of V(II)/V(III) Redox Couple in a Deep Eutectic Solvent: Magnetic Field Effect

**DOI:** 10.3389/fchem.2020.00619

**Published:** 2020-07-21

**Authors:** Rong Cheng, Juncai Xu, Xinyang Wang, Qiang Ma, Huaneng Su, Weiwei Yang, Qian Xu

**Affiliations:** ^1^Institute for Energy Research, Jiangsu University, Zhenjiang, China; ^2^School of Energy and Power Engineering, Jiangsu University, Zhenjiang, China; ^3^School of Science, Jiangsu University, Zhenjiang, China; ^4^School of Energy and Power Engineering, Xi'an Jiaotong University, Xi'an, China

**Keywords:** deep eutectic solvent, redox flow battery, vanadium ions, magnetic field, Lorentz force

## Abstract

Compared with conventional aqueous electrolytes, deep eutectic solvent (DES) has a wider electrochemical stability window, simple preparation, potential biodegradability, and lower cost, leading to its utilization as electrolyte for non-aqueous redox flow batteries (RFB). However, the large viscosity and inferior transport properties hinder the wide spread of DES electrolyte. To circumvent these issues, various additives as well as external fields can be applied separately or synergistically. This work reports a study on the inclusion of a DC magnetic field to the glycol-based DES electrolyte of a RFB. The effects of magnetic field on the physical and electrochemical characteristics of the electrolyte and the active redox couple on mass transfer are studied by cyclic voltammetry and electrochemical impedance spectroscopy. The experimental results show that the viscosity of the vanadium DES electrolyte decreases and the conductivity increases after adding a magnetic field. With the intensity of the added magnetic field increases, the oxidation and reduction peak current densities of the vanadium DES electrolyte keep increasing. Under the magnetic field intensity of 605 mT, the oxidation peak current density and the reduction peak current density increases 41.56 and 30.74%, respectively, compared with those of no added magnetic field. The ohmic resistance and electrochemical reaction resistance of the vanadium DES electrolyte are reduced when adding the magnetic field, reaching to 40.55 and 43.28%, respectively, with a magnetic field intensity of 605 mT. This study shows an effective yet simple way to improve the physical and electrochemical properties of DES electrolyte, which owns the potential to be widely applied in non-aqueous redox flow batteries.

## Introduction

Faced with severe energy and environmental problems, the traditional energy supply structure mainly based on fossil energy can no longer support the sustainable development of human society (Dunn et al., 2011). Therefore, it is necessary to popularize the application of renewable energy, so that renewable energy can gradually convert into dominant energy from auxiliary energy by replacing traditional fossil energy (Xu and Zhao, 2015). Among them, solar energy and wind energy are the ones of the most abundant and easiest to be used, but they have obvious discontinuity, instability and uncontrollablly unstable characteristics, which affects the safe and stable operation of the power grid system (Yang et al., 2011; Xu and Zhao, 2015). Hence developing highly effective and large-scale electrical energy-storage (EES) systems becomes increasingly important to ensure power network stability and reliability (Xu et al., 2018a; Zhang et al., 2018). Redox flow battery (RFB) technology has become one of the most promising energy storage technology due to its flexible design, long charge-discharge cycle life, large energy storage scale, rapid charge-discharge switching response, safety and reliability and environmental benignity (Yang et al., 2011; Xu et al., 2018a; Zhang et al., 2018; Jiang et al., 2019).

In recent years, the researches on RFB technology have experienced a development stage from aqueous system to non-aqueous system. Due to the effect of water decomposition, the electrochemical window of aqueous redox flow battery is narrow (generally <1.5 V). Besides, low solubility and other issues also have limited its application and development (Chen and Hempelmann, 2016; Leung et al., 2017). Therefore, researchers have proposed to use non-aqueous solvents as electrolyte of redox flow batteries. Common non-aqueous solvents are organic solvents (Escalante-García et al., 2014; Wei et al., 2014) and ionic liquids (Abbott and McKenzie, 2006; Zhang et al., 2012; Ejigu et al., 2015), which can provide wide electrochemical windows, but the toxicity, flammability and low solubility of organic solvents and the complex synthesis steps and high cost of ionic liquid synthesis may hinder their large-scale application.

Deep eutectic solvents (DES) are formed when an organic halide salt, typically choline chloride, is combined with a material capable of forming a complex with the halide, such as urea, malonic acid, to form a material that is liquid at ambient conditions (Lloyd et al., 2013; Xu et al., 2018b; Lu et al., 2020). DES can be prepared and used under environmental conditions. In consideration of these appealing features such as a wide electrochemical stability window (Lloyd et al., 2013; Miller et al., 2017), better biocompatibility (Ding et al., 2018; Xu J. et al., 2019), potential biodegradability, low cost, simple preparation procedures, and multi-electron transfer reactions (Zhang et al., 2017), so it is emerged as promising electroactive materials to build sustainable RFBs (Jhong et al., 2009; Zhang et al., 2018).

DESs are hotly exploited in many fields, including metal electrodeposition, chemical reactions, and energy storage (Xu et al., 2018a). There have been preliminary advanced in the application of DES in electrochemical systems. However, compared with aqueous electrolytes, DES has problems such as large viscosity, small diffusion coefficient, and large surface tension, which will cause low power density, serious RFB pumping loss and low energy efficiency (Xu et al., 2018c; Zhang et al., 2018). To address these issues, previous researchers have developed many methods to reduce energy consumption, such as supergravity field (Wang et al., 2010; Liu et al., 2018), intensive electrode arrangement (Pletcher and Li, 2011), magnetic field, ultrasonic vibration (Hung et al., 2012) and many other ways. Compared with many other methods, applying an external magnetic field is more economical and simple method, but few researchers have applied magnetic fields to RFB. In 2015, Wu et al. (2016, 2018) added a magnetic field to a proton exchange membrane battery, which found that the presence of the magnetic field increased the current output density of the battery; Zhu et al. (2016) studied the effect of magnetic field on the transmembrane transport of ions, the permeation of vanadium ions in the vanadium flow battery and the battery performance in 2017. Oz et al. (2016) found that the introduction of a magnetic field during the preparation of lithium battery electrodes, replacing low-spin Co^3+^ with B^3+^, increased the number of high-spin Co^4+^ ions, which makes the lithium battery get more cycles and extend the battery life. Carbone et al. (2017) found that the magnetic field environment introduced in the preparation of sodium battery electrodes significantly increased the reaction activity of the battery electrodes and the working efficiency of the batteries.

Facing the problems of small diffusion coefficient and slow mass transfer of DES electrolyte, in this work, we study the effect of DC magnetic field on the physical and electrochemical properties of vanadium DES electrolytes and active electricity on mass transmission by adding magnetic field of different intensities to the vanadium DES electrolyte of the glycol-based DES flow battery. The continuous Brownian motion of the charged particles in the DES electrolyte of RFB, the forced convection by the mechanical pump and Lorentz force caused by the external magnetic field will cause the charged particles in the electrolyte to change their original motion trajectory. Therefore, their mass transfer process will be changed and ultimately affect the electrochemical reaction. The physical properties of the vanadium DES electrolyte were compared and analyzed based on the test of the viscosity and conductivity of the electrolyte before and after the addition of the magnetic field. The cyclic voltammetry and electrochemical impedance spectroscopy were used to study the effect of the magnetic field on electrochemical kinetics of V(II)/V(III) redox couple. The possible mechanism for the positive effect of the magnetic field was also discussed.

## Experimental

### Preparation of Electrolyte

**Figure 1:**
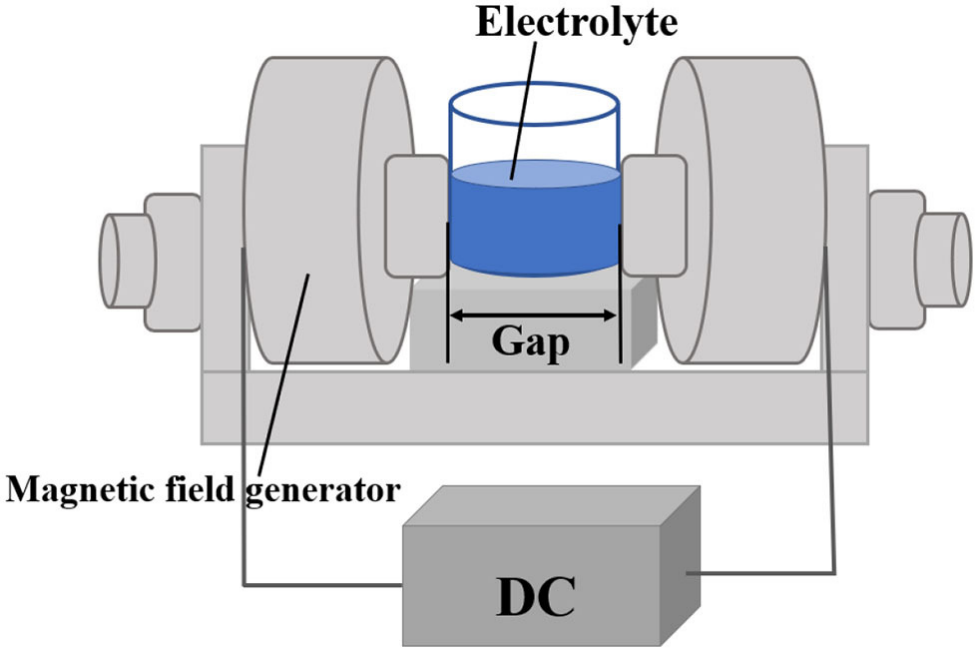
Schematic diagram of the experimental device.

Choline chloride (67-48-1, 98% of Sinopharm Reagent) and ethylene glycol (107-21-1, 99% of Sinopharm Reagent) are mixed at a molar ratio of 1: 2 to prepare DES, and magnetic stirring is performed by constant temperature heating. The device is continuously stirred at a heating temperature of 120°C for 10 min until a colorless and transparent solution is formed. This is called ethaline DES, and it is cooled at room temperature. The configured ethaline DES should be stored in a sealed glass jar in time to prevent the electrolyte from contaminating by the oxygen and water vapor in the air. VCl_3_ (99%, Ourchem) is added to the ethaline DES, and the mixture was heated and stirred at 120°C to prepare a 0.1 M ethylene glycol-based vanadium DES electrolyte. The prepared vanadium DES electrolyte is placed in the magnetic field generator, the magnetic field loading gap is adjusted to 40 mm. By adjusting the current value of the DC power supply connected to the magnetic field generator, the magnetic field intensity of the uniform magnetic field generated by the device is changed. The current value is adjusted from 0 to 8 A, and the corresponding uniform magnetic field intensity is 0 to 605 mT. The specific experimental device diagram is shown in Figure 1.

### Physical Characterizations

#### FT-IR Spectra and Raman Spectra

The electrolytes are tested on a Nicolet Nexus 470 Fourier-transform infrared spectrometer (FT-IR, Thermo Electron, USA) with a resolution of 4 cm^−1^ and a spectral range of 400–4,000 cm^−1^ to obtain the Fourier transform infrared spectrum. Raman spectroscopy are performed on the electrolytes using a laser Raman spectrometer (LRS, Thermo Fisher, USA) with a spectral resolution of <2 cm^−1^. The DES electrolytes with no magnetic field added are used as the initial solution, which are compared with the electrolytes with the magnetic field added.

#### Viscosity and Conductivity Measurements

A digital viscometer (DV-2 + PRO) and a conductivity meter (DDS-307A) are used to test the viscosity and conductivity of DES electrolytes with different magnetic field intensities at room temperature (25 ± 1°C). They are measured three times at each magnetic field intensity, and the average value is recorded.

### Electrochemical Characterizations

Cyclic voltammetry (CV) and electrochemical impedance spectroscopy (EIS) are measured by Chenhua CHI600 electrochemical workstation. These measurements employ a traditional three-electrode system, with a 5 mm diameter graphite electrode as working electrode, a platinum electrode is used as counter electrode and a saturated calomel electrode (SCE) filled with a saturated potassium chloride solution salt bridge is used as reference electrode. Before the test, the DES electrolyte is swept with high purity nitrogen (N_2_, 99.999%) for 20 min to remove all dissolved oxygen. Cyclic voltammetry (CV) scans of vanadium DES electrolytes with different magnetic field intensities are performed in the voltage range of −1.4 to 0.2 V. Each experiment is repeated three times. Meanwhile, in the electrochemical impedance spectroscopy (EIS) measurement, the sinusoidal excitation voltage applied to the battery is 5 mV. The frequency range is set from 0.01 Hz to 100 kHz.

## Results and Discussion

### Physical Characteristics of Vanadium Ions in Ethaline DES

#### FT-IR Spectra Test and Raman Spectra Test

**Figure 2:**
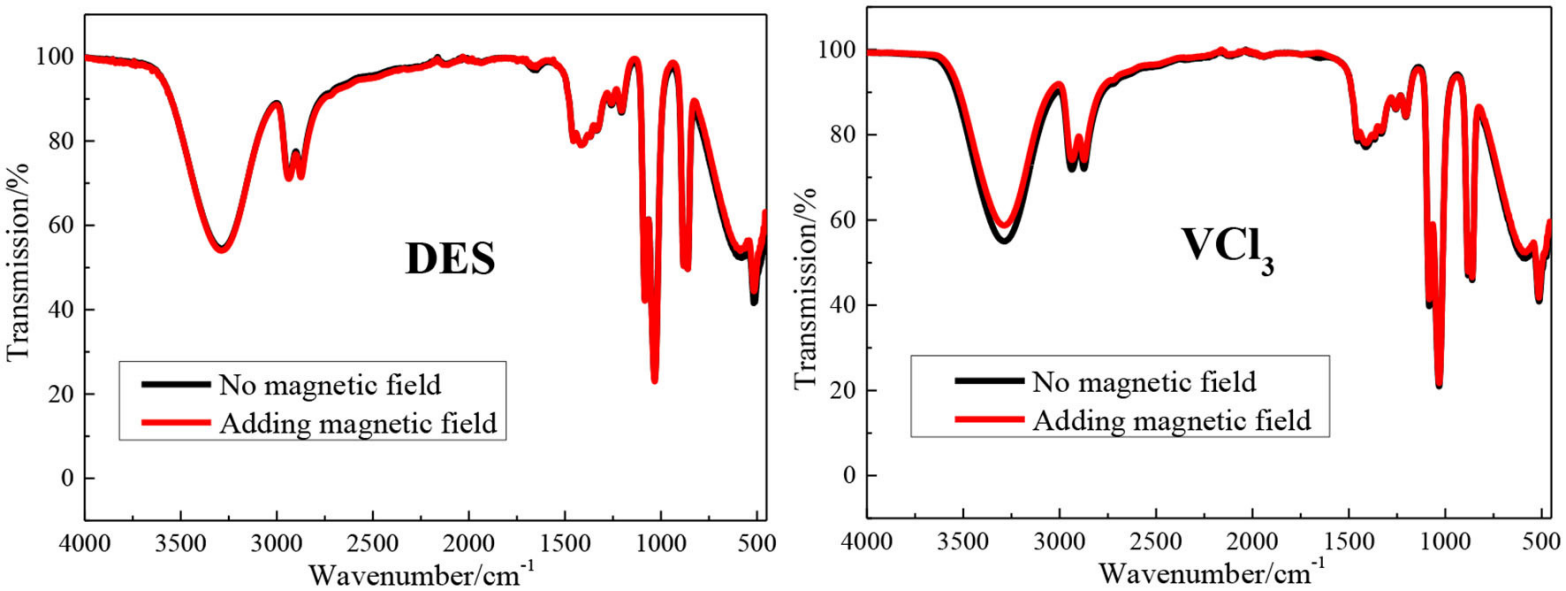
The FT-IR spectra of blank ethaline DES electrolyte and vanadium DES electrolyte with or without magnetic field.

**Figure 3:**
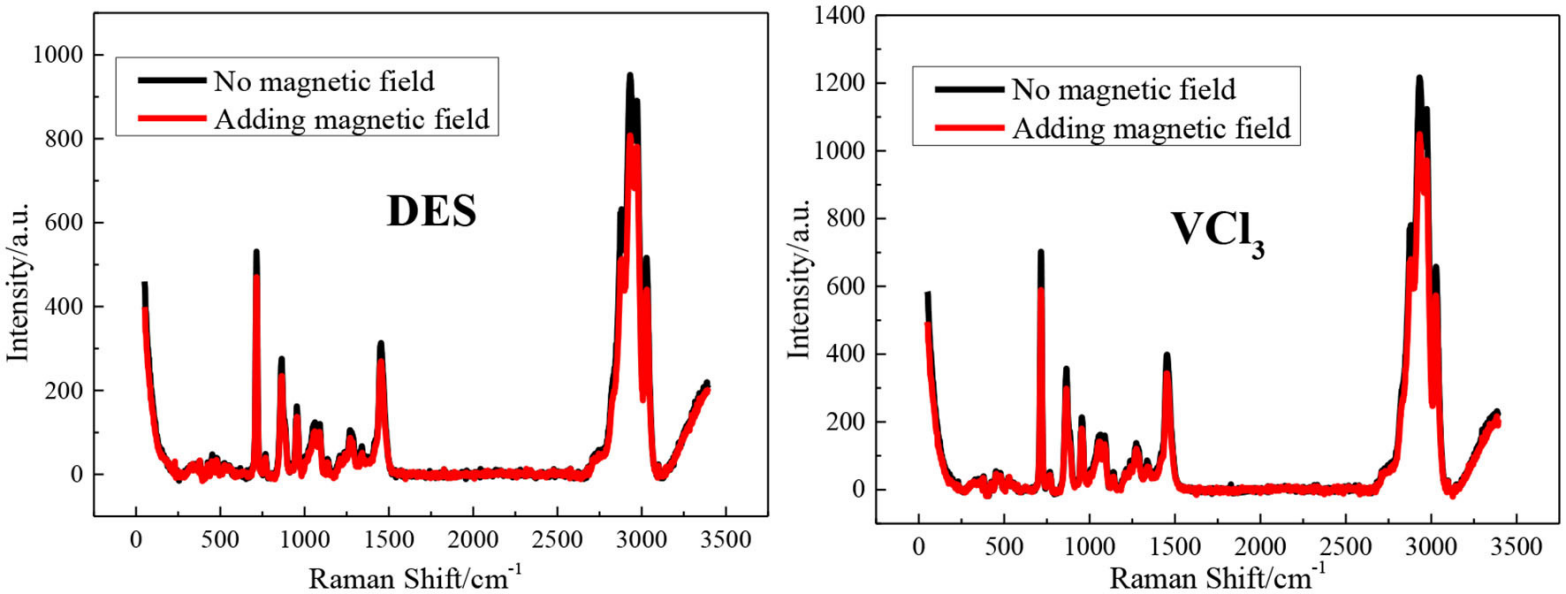
The Raman spectra of blank ethaline DES electrolyte and vanadium DES electrolyte with or without magnetic field.

The FT-IR spectrum of the blank ethaline DES electrolyte and vanadium DES electrolyte with and without magnetic field are shown in Figure 2. All tests are at room temperature (25 ± 1°C). The band at 3,345 cm^−1^ is due to O–H stretching vibration; the peaks at 2,950 and 2,875 cm^−1^ are due to the stretching vibration peak of –CH_3_ or –CH_2_ in choline chloride; The peak at 3,029 cm^−1^ is the result of the eutectic mixture of choline chloride and ethylene glycol; the peaks found at 954 and 1,041 cm^−1^ are the stretching vibration of C–C in choline chloride Peak and ethylene glycol C–O stretching vibration peak (He et al., 2015; Xu et al., 2015). The blank ethaline DES electrolyte and vanadium DES electrolyte FT-IR spectrum characteristic peaks before and after the magnetic field addition are basically the same, and no new characteristic peaks appear, indicating that the electrolytes composition after the magnetic field addition are stable, and no decomposition or new substances appear. Then the Raman spectra test are performed on the electrolytes added with magnetic field for further analysis and research, as shown in Figure 3. By examining the Raman spectra of choline chloride and ethylene glycol (Wang et al., 2014), it can be found that the characteristic peaks at 717, 869, 957, 1,453 cm^−1^ and 1,093, 1,271, 1,464 cm^−1^ belong to choline chloride and ethylene glycol, respectively, and the characteristic peaks appearing around 3,000 cm^−1^ are the result of the superposition of the two characteristic peaks. From the graph, there is no new absorption peak or conspicuous peak shift in the electrolytes after adding the magnetic field. Accordingly, it is preliminarily determined that the addition of the magnetic field does not transform the chemical reaction of the ions inside the electrolyte to form new substances. Through FT-IR spectrum and Raman spectrum, the results demonstrate that the addition of magnetic field will not transform the composition and molecular structure of the vanadium DES electrolyte.

#### Conductivity and Viscosity of Ethaline DES Electrolyte With Vanadium Ions

**Figure 4:**
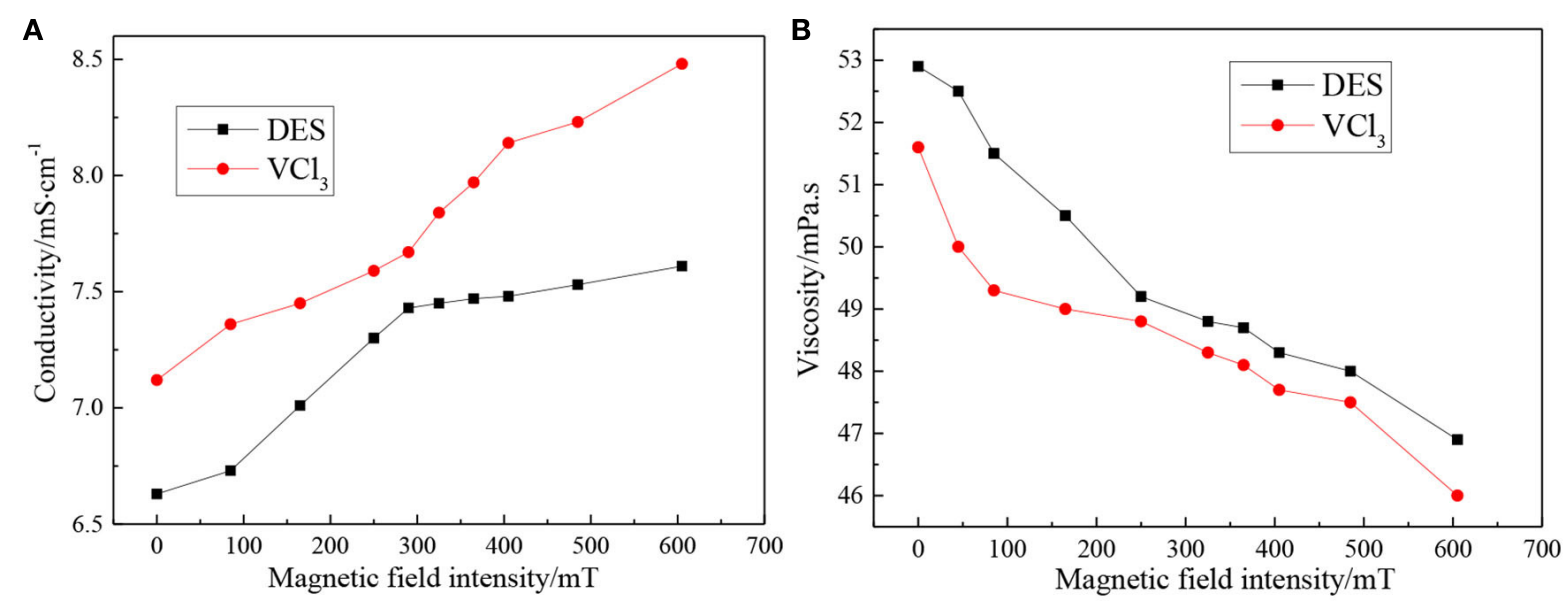
**(A)** Conductivity and **(B)** viscosity of blank ethaline DES electrolyte and vanadium DES electrolyte under different magnetic field intensities.

The conductivity and viscosity of blank ethaline DES electrolyte and vanadium DES electrolyte with different magnetic field intensities at room temperature (25 ± 1°C) are shown in Figure 4. Figure 4A shows the conductivity of the two electrolytes as the magnetic field intensities changes. This figure illustrates that adding magnetic field can improve the conductivity of the electrolytes, and as the intensity of the magnetic field increases, the conductivity of the electrolytes will further increase. It is worth mentioning that the conductivity of the vanadium DES electrolyte without the addition of magnetic field is 7.12 mS·cm^−1^. As the magnetic field intensity increases, its conductivity increases to 8.48 mS·cm^−1^. This result indicates that the addition of magnetic field can enhance the ability of electrolyte to conduct charge and improve the conductivity of the electrolyte. In addition, the conductivity of the electrolyte is related to its viscosity. By testing the viscosity of the electrolytes under different magnetic field intensities, it can be seen from Figure 4B that the viscosity of the electrolytes decreases with the increase of the magnetic field intensity. The viscosity of the vanadium DES electrolyte is reduced from 51.6 to 46 mPa·s. According to the hole theory (Fürth, 1941; Abbott, 2004), the viscosity of the system is controlled by the mobility of ions and the utilization rate of holes at the appropriate scale. By enhancing the intensity of the magnetic field, the Lorentz force of the ions in the electrolyte is increased, the movement of the ions is increased, and the spacing is increased, which will generate more holes between the ions, hence the free moving space increases, the fluidity increases, and the viscosity decreases. On the other hand, the magnetic field accelerates the movement speed of the conductive ions and increase the kinetic energy, thus weakening the force between different charges and the intermolecular association. Therefore, the resistance to ion motion reduces and the viscosity decreases.

#### Diffusivity of Vanadium Ions in Ethaline DES Electrolyte

**Figure 5:**
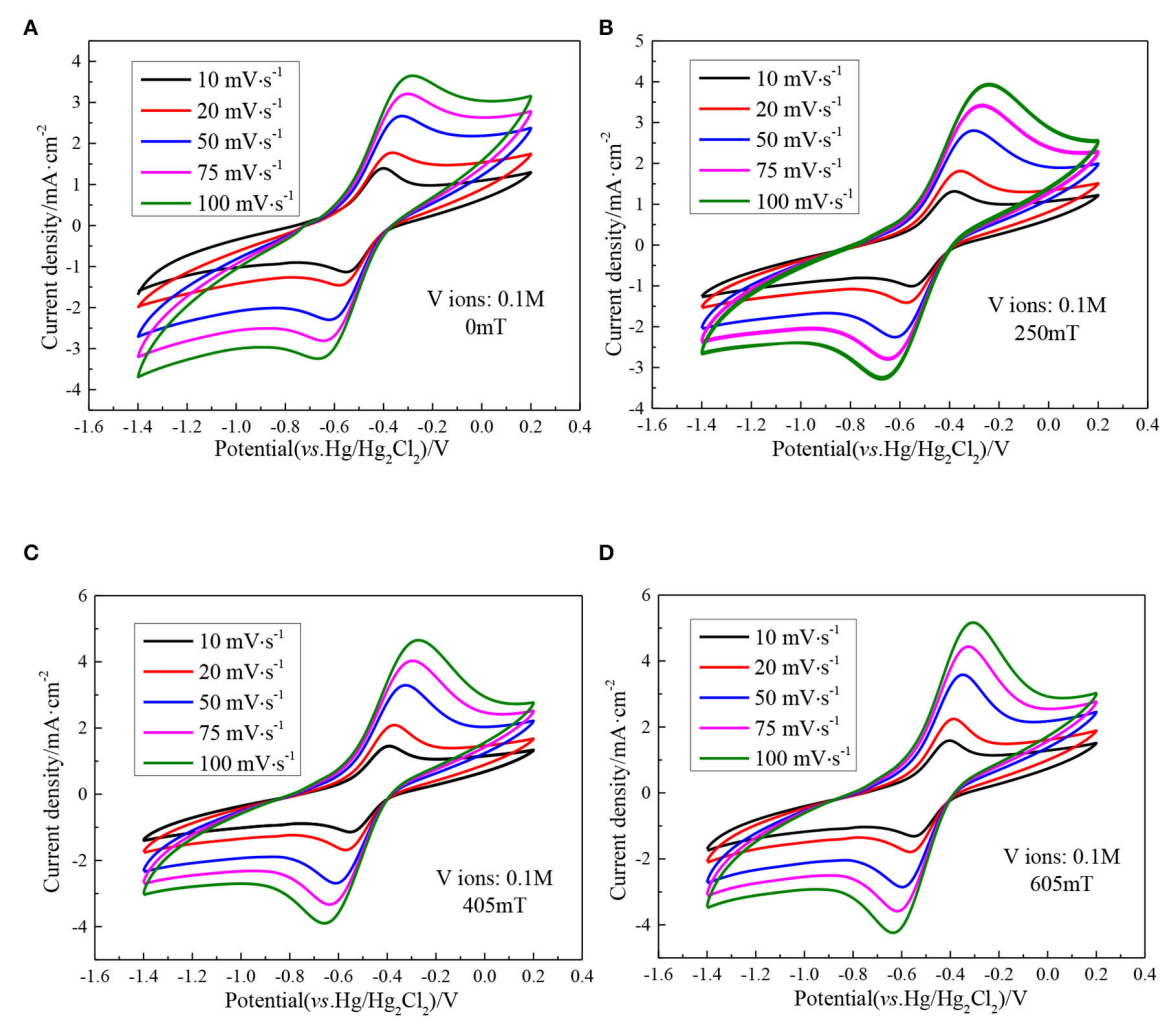
CV curves of 0.1 M VCl_3_ with different magnetic field intensities at different scan rates, **(A)** 0 mT; **(B)** 250 mT; **(C)** 405 mT; **(D)** 605 mT.

Figure 5 indicates a series of CV curves of the ethaline DES electrolyte containing 0.1 M vanadium ions with different magnetic field intensities at different scan rates. It can be seen from the figure that as the scan rate increases, the peak potential difference of the vanadium DES electrolyte continuously increases. In the absence of a magnetic field the peak potential difference Δ*E* is 161 mV at a scan rate of 10 mV·s^−1^, and increases to 391 mV at a scan rate of 100 mV·s^−1^. When the magnetic field strength of 605 mT is added, it is 149 mV at a scan rate of 10 mV·s^−1^ and increases to 333 mV at a scan rate of 100 mV·s^−1^. The decrease of the peak potential difference by adding magnetic field indicates that under high magnetic field intensities, the reversibility of redox reaction is enhanced. In addition, with the increase of the scan rate, the ratio of the oxidation peak current to the reduction peak current remained basically close to 1, indicating that the V (II)/V (III) redox couple showed quasi-reversible electrochemical kinetics. For the quasi-reversible nature of V (III)/V (II) redox reaction, the diffusion coefficient of V (III) ions can be calculated according to the Randles-Sevcik equation.

For reversible reactions (Bard and Faulkner, 2002):

(1) $$\begin{array}{l} i_{p}=2.69\times 10^{5}An^{1.5}cD^{0.5}v^{0.5} \end{array}$$

For irreversible reactions (Casas et al., 2005):

(2) $$\begin{array}{l} i_{p}=2.99\times 10^{5}An^{1.5}\alpha^{0.5}v^{0.5} \end{array}$$

Where *i*_*p*_ is the peak current (A), *n* is the number of electrons involved in the electrode reaction, α is the charge transfer coefficient, *A* is the electrode area (cm^2^), *c* is the concentration of the active material (mol·cm^−3^), and *D* is the diffusion coefficient (cm^2^·s^−1^), and ν is the scan rate (V·s^−1^). The charge transfer coefficient α can be obtained by the following formula (Xu et al., 2015; Xu J. C. et al., 2019):

(3) $$\begin{array}{l} {\left|E_{p}-E_{p/2}\right|}_{irrev}=\frac{47.7mV}{\alpha } \end{array}$$

*E*_*p*_ and *E*_*p*/2_ represent the potential of the peak current density and the potential of the half-peak current density, respectively.

**Figure 6:**
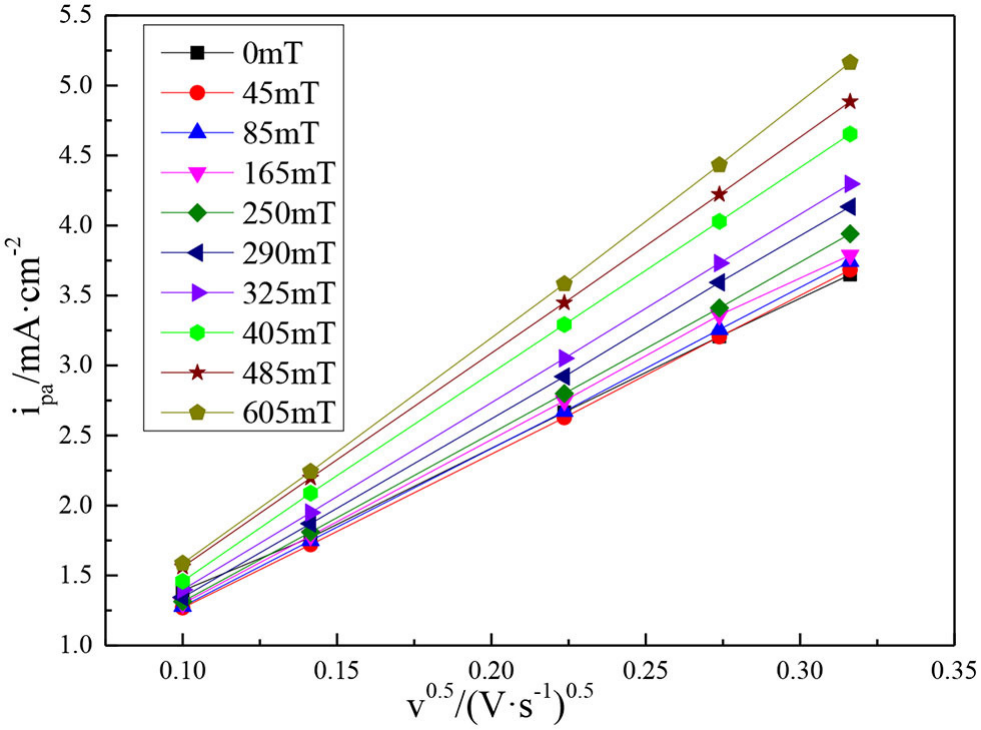
Plots of peak current density vs. the square root of scan rates under different magnetic field intensities.

Figure 6 shows the relationship between the anodic peak current density (*i*_*pa*_) of the vanadium DES electrolyte and the square root of the scan rate under different magnetic field intensities. It can be intuitively found that the peak current density increases linearly with the increase of the scan rate, which indicates that the redox reaction of vanadium DES electrolyte is still under diffusion control after the magnetic field is added. Because the V(III)/V(II) redox reaction is quasi-reversible, its diffusion coefficient is between reversible (*D*_*re*_) and irreversible (*D*_*irre*_). The diffusion coefficients of vanadium ions estimated from Equations (1) and (2) are listed in Table 1. It can be seen from Table 1 that there is a positive proportional relationship between the magnetic field intensity and the diffusion coefficient. The diffusion coefficient of the vanadium ion under the 605 mT magnetic field intensity is calculated in the range of 3.683 × 10^−7^~1.050 × 10^−6^ cm^2^s^−1^, which is much higher than the original electrolyte (1.838 × 10^−7^~5.614 × 10^−7^ cm^2^s^−1^). Numerically, the diffusion coefficient increases by an order of magnitude, illustrating that the addition of magnetic field affects the electrochemical reaction of vanadium redox couples and enhances the mass transfer capacity of vanadium ions. Therefore, the calculation results present that the addition of magnetic field can promote mass transfer and component diffusion in the electrolyte, which promote redox reaction.

**Table 1:** Diffusion coefficient of vanadium ions at different magnetic field intensities.

**Magnetic field** **intensity (mT)**	**0**	**85**	**165**	**250**	**325**	**405**	**605**
*D_*re*_* × 10^−7^(cm^2^·s^−1^)	1.838	1.935	1.981	2.145	2.550	2.991	3.683
*D_*irre*_* × 10^−7^(cm^2^·s^−1^)	5.614	6.863	6.959	7.207	8.525	9.490	10.50

### Electrochemical Characteristics of Vanadium Ions in Ethaline DES

#### Cyclic Voltammetry

**Figure 7:**
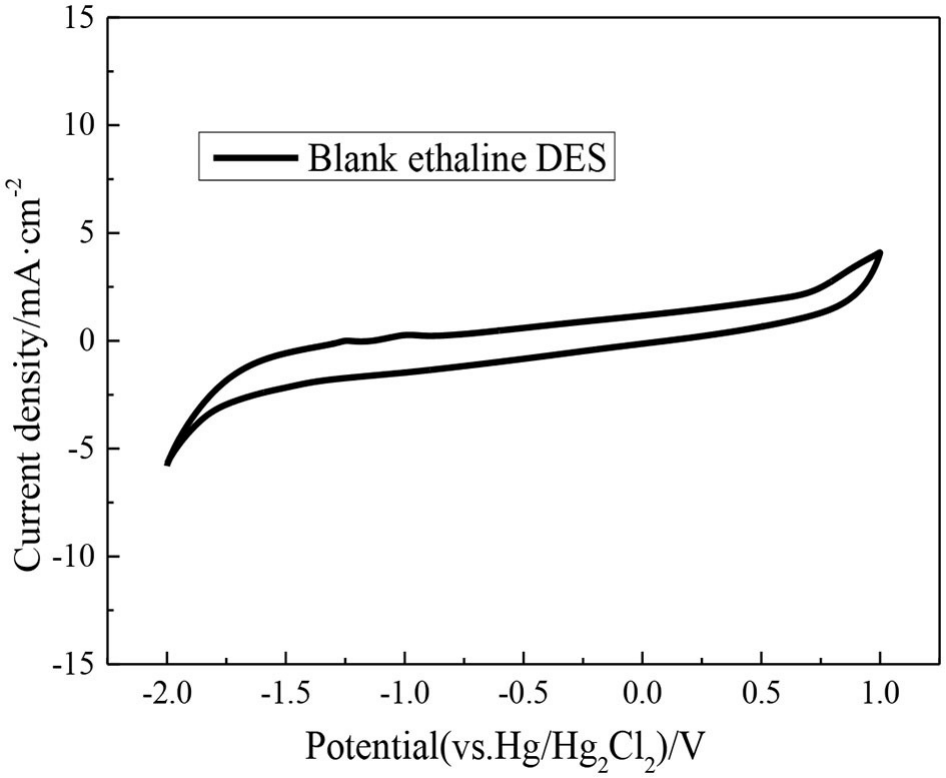
CV curve of blank ethaline electrolyte.

Before studying the electrochemical characteristics of the V(II)/V(III) redox reaction, blank ethaline DES electrolyte cyclic voltammetry is tested at room temperature. The scan voltage range from −2.0 to 1.0 V (relative to Hg/Hg_2_Cl_2_). The CV curve obtained in the experiment is shown in Figure 7. There is no oxidation or reduction reaction peak in this voltage range, which can be seen that ethaline DES as the electrolyte has a wider and more stable electrochemical window compared with the traditional aqueous electrolyte.

**Figure 8:**
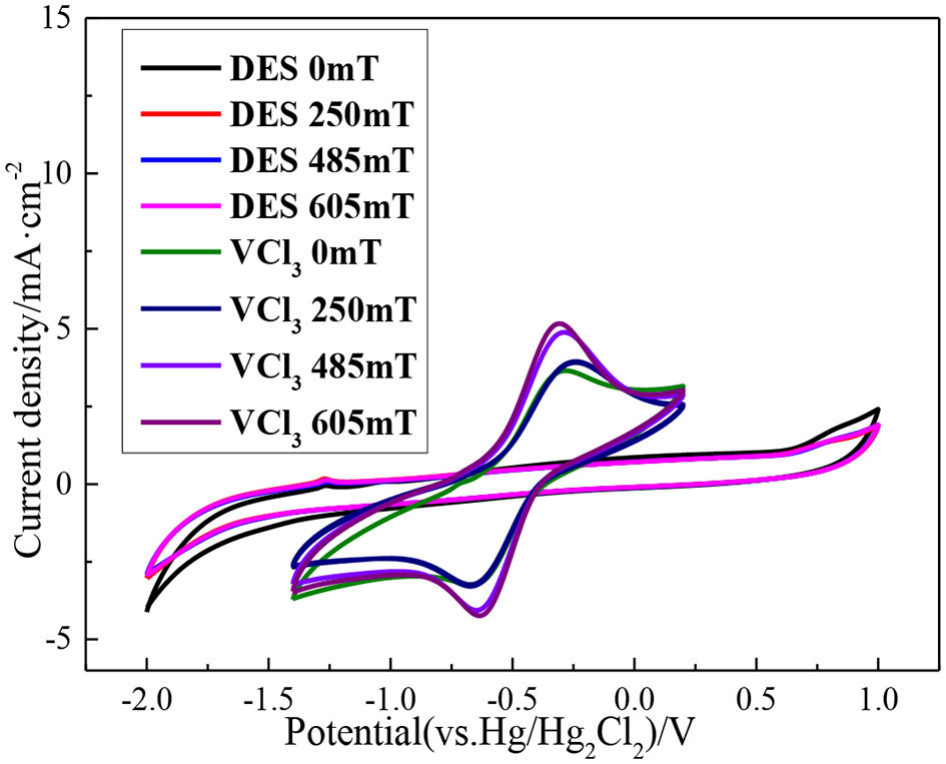
CV curves of blank ethaline DES electrolyte and vanadium DES electrolyte under different magnetic field intensities.

The blank ethaline DES electrolyte is first added with magnetic fields of different intensities, and the CV scanning test is carried out in the voltage range −2.0 to 1.0 V. Secondly, the ethaline DES electrolyte containing 0.1 M VCl_3_ is added with magnetic fields of different intensities. And the CV scanning test is carried out at multiple scanning rates within the voltage of −1.4 to 0.2 V. Before anatomizing the effect of different magnetic field intensities on the vanadium DES electrolyte, it is necessary to study the effect of different magnetic field intensities on the blank ethaline DES electrolyte, so that the target of the magnetic field can be ascertained. The CV curves of blank ethaline DES electrolyte and vanadium DES electrolyte under different magnetic field intensities are shown in Figure 8. It can be seen from the figure that different magnetic field intensities have a slight effect on the blank ethaline DES electrolyte near −2.0 and 1.0 V. When the potential range is −1.4 to −0.2 V, the magnetic field has basically no effect on the blank ethaline DES electrolyte. But the CV curve of vanadium DES electrolyte in this range represents that with the increase of magnetic field intensity, the CV curve has apparent change, so it can be known that the magnetic field has a significant effect on vanadium ion in vanadium DES electrolyte.

**Figure 9:**
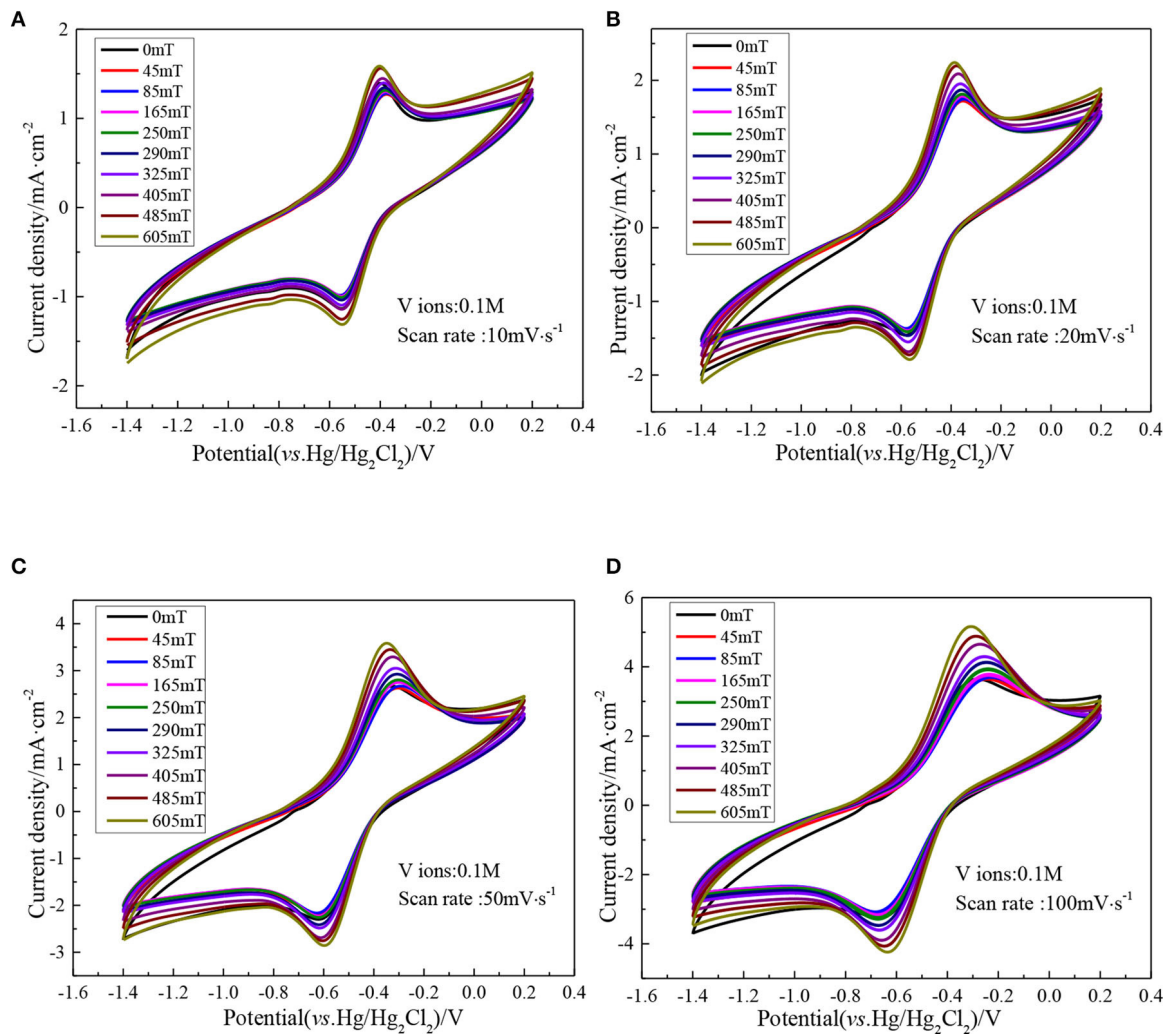
CV curves of 0.1M VCl_3_ adding different magnetic field intensities at different scan rates, **(A)** 10 mV·s^−1^; **(B)** 20 mV·s^−1^; **(C)** 50 mV·s^−1^; **(D)** 100 mV·s^−1^.

In the range of 0–605 mT magnetic field intensity, the CV curve of vanadium DES electrolyte is shown in Figure 9, which shows a pair of distinct redox peaks, corresponding to the V (III)/V (II). Additionally, there is no new redox peak is generated, which further confirms that the addition of magnetic field does not result in other chemical reactions of vanadium ions to form new substances, and also show that the redox reaction of the active substance is not changed. In order to intuitively see the effect of magnetic field on electrolyte, the peak current density at different scan rates is listed in Table 2. Taking the scan rate of 100 mV·s^−1^ as an example, the redox peak current densities of the original electrolyte are 3.65 and −3.25 mA·cm^−2^, respectively. When magnetic field is added, the redox peak current densities have all increased, and reach a maximum when the magnetic field intensity is 605 mT, which is 5.16 and −4.24 mA·cm^−2^, respectively. This indicates that the introduction of magnetic field causes the ions to be affected by Lorentz force, and increases the ionic diffusion ability in the course of electrochemical reaction, which accelerates the electrochemical reaction rate of ions, accelerates the movement of the ions in the solution, and then increases the collision between ions to make it easier to overcome the activation energy and electrochemical reaction. In addition, the difference of electrochemical potential between the forward oxidation peak and the backward reduction peak of the vanadium ions in the vanadium DES electrolyte decrease with the increase of magnetic field intensity. Taking the sweep speed of 100 mV·s^−1^ as an example, the peak potential difference Δ*E* without magnetic field is 391 mV. When the magnetic field intensity of 605 mT is added, it decrease to 333 mV. This indicates that the reversibility of V(II)/V(III) is enhanced at high magnetic field intensity.

**Table 2:** Peak current density at different scanning rates.

**Magnetic field** **intensity (mT)**	**Oxidation peak current density** **(mA·cm**^**−2**^**)**				**Reduction peak current density** **(mA·cm**^**−2**^**)**			
	**10** **mV·s^**−1**^**	**20** **mV·s^**−1**^**	**50** **mV·s^**−1**^**	**100** **mV·s^**−1**^**	**10** **mV·s^**−1**^**	**20** **mV·s^**−1**^**	**50** **mV·s^**−1**^**	**100** **mV·s^**−1**^**
0	1.39191	1.77235	2.66515	3.64809	−1.13318	−1.4576	−2.29489	−3.24269
165	1.29921	1.78355	2.74816	3.78814	−0.99415	−1.39954	−2.22511	−3.19787
250	1.31398	1.80953	2.80011	3.94093	−1.00586	−1.41126	−2.25669	−3.29158
325	1.39802	1.94958	3.05068	4.29744	−1.0955	−1.5503	−2.48434	−3.59868
605	1.58595	2.24243	3.5829	5.16426	−1.31296	−1.78916	−2.85511	−4.23938

#### Electrochemical Impedance Spectroscopy

**Figure 10:**
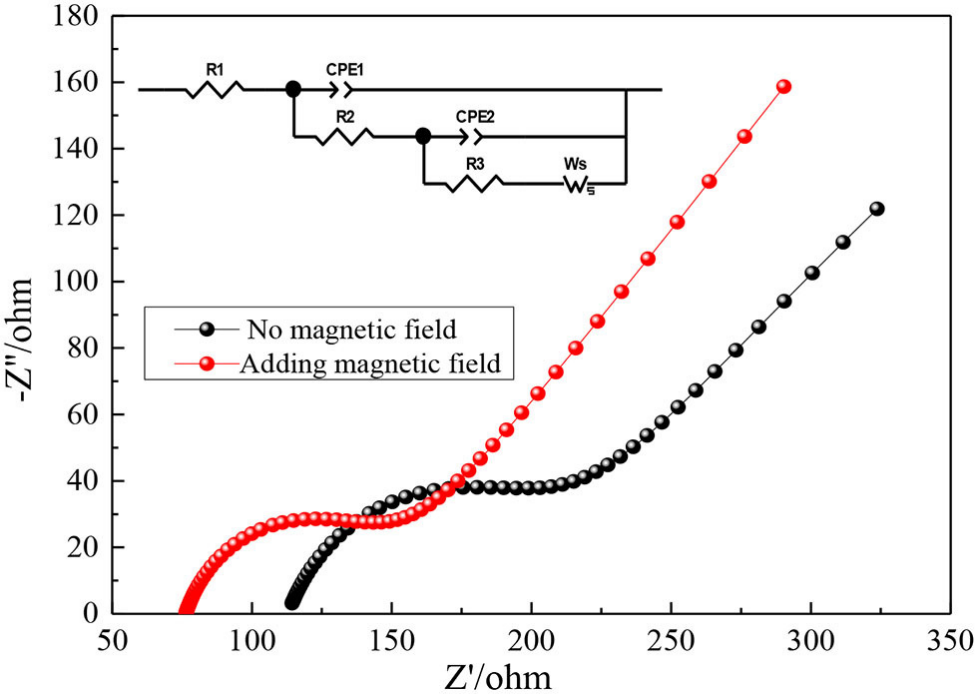
Nyquist plot of 0.1 M VCl_3_ electrolyte with or without magnetic field.

The influence of magnetic field on the electrochemical performance of V (III) DES electrolyte is further analyzed by electrochemical impedance spectroscopy (EIS). The corresponding equivalent circuit diagram is shown in the upper left corner of Figure 10. The meanings of each electrochemical parameter in the figure are as follows: R1 represents the resistance of the ion migration process in the solution, that is, the ohmic resistance of the solution. R2 stands for the electrochemical reaction resistance. R3 and Ws are the concentration polarization resistance, which simulates the mass transfer of liquid phase. CPE is the capacitor element, simulating the charge and discharge process. The Nyquist diagram of vanadium electrolyte without magnetic field and magnetic field is shown in Figure 10. Each curve in the figure shows a similar law: a single concave semicircle in the high frequency region and a straight line in the low frequency region, which indicates that the redox reaction of V(III)/V(II) is controlled by the mixture of electrochemical reaction and diffusion step (Shen et al., 2015; Lu et al., 2019). The semicircular part corresponds to the transfer reaction at the electrode-electrolyte interface, while the linear part is relevant to the diffusion of vanadium species in solution (Yao et al., 2012).

It can be seen from Figure 10 that after adding a magnetic field to the electrolyte solution, the ohmic resistance R1 and the electrochemical reaction resistance R2 of the solution are reduced. The fitting data is obtained by using Z-SimpWin software. From the values in Table 3, it can be seen intuitively that the R1 of the DES electrolyte solution with magnetic field is reduced from the original 112.91 to 67.13 Ω, and R2 is reduced from the original 101.34 to 57.48 Ω. The reason for the decrease of the ohmic resistance in the solution may be the movement of ions in the electrolyte under the action of the magnetic field is enhanced, which enhances the DES electrolyte fluidity, reduces the viscosity, and increases the conductivity. Decreasing the electrochemical reaction resistance indicates that the charge transfer process of the electrolyte is accelerated, which manifests the higher electrochemical reaction rate. This should be the charged particles of the electrolyte are affected by Lorentz force with magnetic field. These results further confirm that the V(III)/V(II) redox reaction can be improved by adding magnetic field, which is consistent with the CV results.

**Table 3:** The parameters of 0.1 M VCl_3_ electrolyte impedance obtained from fitting the EIS plots with the equivalent circuit.

**Working condition**	**R1 (Ω)**	**R2 (Ω)**
No magnetic field	112.91	101.34
Adding magnetic field	67.13	57.48

### Mechanism Discussion

In view of the above results, it is shown that the magnetic field has an influence on vanadium ion diffusion, electrode-electrolyte interface behavior, electrochemical reaction rate, and electrolyte impedance. From the quantum theoretical analysis of chemical reactions, chemical changes depend on the electronic spins of reactive particles. It is precisely the effect of the magnetic field effect on the chemical reaction is reflected by affecting the spin properties of the electron (Cheng, 1989). In the course of the process of electrochemical reactions, there are electrons delivery and transfer, and ions movement. Since the moving charged particles are affected by the Lorentz force generated in the magnetic field, the motion trajectory is changed. Therefore, the magnetic field will affect the electrochemical reaction to a certain extent. From the nature of electrochemical reactions, the fundamental factor of magnetic fields is that the magnetic field environment can change the spin direction of electrons, the properties of the spin, the energy of the spin, and the phase of the spin, which transforms the entropy of the reaction system and determines the chemical properties of charged particles (Jiang and Yan, 1992; Ma et al., 2019).

From the perspective of magnetic bond theory (Cheng, 1989; Hu et al., 2000), there are universal magnetic bonds in the structure of matter. The so-called magnetic bond refers to the strong attraction between the magnetic N-pole wave and the magnetic S-pole wave produced by the particles (such as electrons and protons) constituting the substance. Due to electricity and magnetism are the inseparable unity, each charged particle can be regarded as a magnet with magnetic N pole and magnetic S pole. A magnetic bond is formed by the interaction between different magnetic poles. The direction, strength, or amount of residual bonding of magnetic bonds determines the process and result of chemical reactions, as well as the variety of chemical reactions. When the magnetic bond is subjected to the magnetic field, its direction and arrangement will be changed, thereby affecting the generation and progress of chemical reactions.

In this work, the flow of the electrolyte is changed due to the Lorentz force by applying the magnetic field to the vanadium ion electrolyte, and the trajectory of the vanadium ions movement is also changed. As a consequence, the concentration polarization is reduced, and the vanadium ion diffusion capacity is increased, which eventually accelerates the electrochemical reaction. Theoretically, these phenomena indicate that under the influence of the magnetic field, the ions are affected by the Lorentz force, the movement rate is accelerated, and the kinetic energy is increased, thereby weakening the force between the different sign charges, making it easier for the ions to overcome the hydrogen bonding between the ions and the polymerization effect (Su et al., 2017, 2018), the probability of collision between ions and ions increases, and its intermolecular association and interionic association is weakened. In addition, when the magnetic field acts on the DES electrolyte, it may make the single molecule state dominant, weaken the association between molecules, reduce the ability to form hydrogen bonds between molecules in the DES electrolyte and reduce the number of hydrogen bonds (Qiang et al., 2019), thereby affecting the electrochemical reaction of the electrolyte. Macroscopically speaking, the Lorentz force caused by externally applied magnetic field induces the magnetohydrodynamic effect (MHD), which can promote the convective motion of the DES electrolyte, promote the components diffusion and mass transfer in the DES electrolyte, and ultimately increase the mass transfer speed. Combined with the magnetic bond theory to explain this phenomenon, the charged particles in DES electrolyte are regarded as several small magnets, which are bonded by magnetic bonds. Obeying the Pauli incompatibility principle, the spin directions of any paired electrons are in opposite directions. Unpaired electrons can freely move in any direction under the effect of magnetic bonds. When the external magnetic field is applied, the magnetic bonds of these unpaired electrons tend to be in the same direction as the external magnetic field, which changes the distribution and arrangement of charged particles in the electrolyte, which further changes the entropy of the reaction system and then affects the electrochemical reaction progress. It is found through experiments that the diffusion coefficient of vanadium ions increases with the increase of magnetic field intensity. The reason is that the Lorentz force has a stirring effect on vanadium ions in the electrolyte, which makes the mobility of vanadium ions in the DES electrolyte enhanced (Qiang et al., 2019).

## Conclusion

In this work, the physical and electrochemical characteristics of the V(III)/V(II) redox couple in a glycol-based DES electrolyte under the inclusion of an external magnetic field are studied. The viscosity, conductivity, CV curve and electrochemical impedance spectroscopy of vanadium ion ethaline electrolyte before and after adding 0–605 mT magnetic field intensities are measured, respectively. The experimental results demonstrate that the addition of magnetic field enhances the ability of electrolyte to conduct charge, reduces the viscosity of the electrolyte and thus reduces unnecessary energy loss. Cyclic voltammetry curves prove that the addition of magnetic field can increase the redox peak current density of the electrolyte, accelerate the redox reaction rate of V(III)/V(II) and enhance the reversibility of V(II)/V(III). From the analysis of electrochemical impedance spectroscopy, the ohmic resistance and electrochemical reaction resistance of the electrolyte decrease after the magnetic field is added, which indicates that the charge transfer process is accelerated and reflects the higher electrochemical reaction rate, caused by the Lorentz force on charged particles. This study discloses that adding magnetic field to the glycol-based DES electrolyte can improve its transport properties thus play a positive role in the mass transfer process, and improve the reaction kinetics of the V(III)/V(II) redox couple. The effect of the magnetic field on the working performance of the non-aqueous DES electrolyte redox flow battery is still ongoing.

## Data Availability Statement

The raw data supporting the conclusions of this article will be made available by the authors, without undue reservation.

## Author Contributions

RC: carrying out experiments, analysis, and writing original draft. JX: carrying out experiments and analysis. XW: methodology and analysis. QM: conceptualization. HS: resources and investigation. WY: methodology and resources. QX: conceptualization, funding acquisition, project administration, writing—review and editing, and supervision. All authors contributed to the article and approved the submitted version.

## Conflict of Interest

The authors declare that the research was conducted in the absence of any commercial or financial relationships that could be construed as a potential conflict of interest.
